# Baricitinib in pediatric chronic immune thrombocytopenia and associated autoimmune conditions: a case report

**DOI:** 10.3389/fped.2024.1516039

**Published:** 2024-12-24

**Authors:** G. López de Hontanar Torres, J. Zubicaray, E. Sebastián, A. Hernández-Martín, J. Iriondo, J. Sevilla

**Affiliations:** ^1^ Pediatric Hematology and Oncology Department, Hospital Infantil Universitario Niño Jesús, Madrid, Spain; ^2^ Dermatology Department, Hospital Infantil Universitario Niño Jesús, Madrid, Spain

**Keywords:** immune thrombocytopenia, platelet disorders, adolescent, baricitinib, alopecia areata

## Abstract

Immune thrombocytopenia (ITP) is a disease characterized by platelet destruction, presenting substantial challenges in clinical practice. The classic first line therapeutic management includes corticosteroids and intravenous immunoglobulins. Although it is less frequent in children than in adults, there is a significant percentage of patients, up to 47% according to the Pediatric and Adult Registry on Chronic ITP, who require second-line or further treatment, due to non-response to the first line treatment or persistence of disease, among other reasons. Several second line approaches for its treatment are currently in use, including increasing platelet production with thrombopoietin receptor agonists. We report the case of a 16-year-old patient with ITP and alopecia areata successfully treated with baricitinib, a reversible and selective JAK 1/2 inhibitor. Baricitinib is currently in use for the treatment of several autoimmune conditions and has been shown to increase platelet counts in these patients. This phenomenon has been linked to increased TPO signaling and reduced platelet destruction. There are promising preliminary results of adult ITP patients treated with baricitinib. This case report is the first reported use of baricitinib in ITP in the pediatric and adolescent setting, potentially leading to its use in this condition.

## 1 Background

Immune thrombocytopenia (ITP) is an autoimmune disorder characterized by accelerated platelet destruction, leading to a decrease in platelet count. Its unpredictable course, variable response to treatment, and potential for life-threatening bleeding complications represent a significant challenge. Conventional first line therapeutic approaches such as corticosteroids or immunoglobulins have shown significant efficacy but often come with considerable adverse effects. However, according to the Pediatric and Adult Registry on Chronic ITP (cITP), between 38% and 47% of pediatric patients may require a second-line therapy after the first 6 months of treatment (1), such as thrombopoietin receptor agonists.

Baricitinib is a Janus kinase (JAK) 1/2 inhibitor, used in the treatment of rheumatoid arthritis, atopic dermatitis, and alopecia areata (2), with potential use in several other autoimmune conditions. A transient increase in platelet counts has been observed in patients treated with baricitinib. Therefore, it may have potential use in the treatment of thrombocytopenia.

## 2 Case presentation

We report the first case of a pediatric patient with cITP and alopecia areata successfully treated with baricitinib: A 16-year-old woman consulted in the emergency room for asthenia of 2 months duration, which worsened during the last 15 days before consulting. She also reported increased menstrual bleeding in the last 2 months as well as hair loss. The patient reported a history of atopic dermatitis and a familial history of acute myeloid leukemia.

She had general paleness, petechiae in lower limbs and a 3 cm patch of alopecia on her scalp, with no other significant findings on physical examination.

Blood tests (Table 1) showed a marked microcytic anemia, high reticulocyte count and severe thrombocytopenia of 6 × 10^9 ^/L. The peripheral blood smear showed left shifted myeloid cells, and microcytic and hypochromic erythrocytes, with no evidence of platelet clumping or other platelet abnormalities. A ferritin level of 8.60 ng/ml was noted, consistent with iron deficiency anemia, as well as a moderate increase in liver enzymes. There were no other significant findings. She was admitted to the Hematology ward for evaluation.

**Table 1:** Complementary tests at diagnosis.

Complete Blood Count	Eythrocytes	3.31 × 10^12^/L (4.00 × 10^12^–5.30 × 10^12^/L)
	Leucocytes	7.5 × 10^9^/L (4.5 × 10^9^–11 × 10^9^/L)
	Neutrophils	5.03 × 10^9^/L (1.5 × 10^9^–8.0 × 10^9^/L)
	Hemoglobin	660 g/L (110–156 g/L)
	MCV	71.1 fL (82.5–96.5 fL)
	MCHC	199 g/L (290–340 g/L)
	Platelets	7 × 10^9^/L (150 × 10^9^–400 × 10^9^/L)
	Mean platelet volume	9.8 fL (5.0–10.0 fL)
	Reticulocytes	160 × 10^9^/L (24 × 10^9^–84 × 10^9^/L)
Hemolysis parameters.	Total bilirubin	0.39 mg/dL (0.20–1.30 mg/dL)
	LDH	251 U/L (100–250 U/L)
	Haptoglobin	42 mg/dL (30-200 mg/dL)
Direct Coombs Test	Negative	
Peripheral blood smear	Anisocytosis, microcytosis and hypochromia. 4 erythroblasts per 100 leucocytes. Left-shifted granulocytes.	
Inmunoglobulines	IgG	990 mg/dl (623–1584 mg/dL)
	IgA	50.2 (40–350 mg/dL)
	IgM	87 (50–300 mg/dL)
Autoinmune screening	Anti-tissue transglutaminase antibodies	0.1 U/ml (<3.5 U/ml)
	Anti-microsomal antibodies	8547 U/ml (5–60 U/ml)
	Antinuclear antibodies	Negative
	Complement C3	104 mg/dl (91–163 mg/dl)
	Complement C4	17.6 mg/dl (13–43 mg/dl)
	Rheumatoid factor	<2 UI/ml (<14 UI/ml)
	Anti Ro-52 antibody	Negative
	Antiphospholipid antibodies	Negative
Microbiological studies	Cytomegalovirus	Positive IgG, negative IgM
	Epstein-Barr	Positive IgG, negative IgM
	Herpes simplex 1 & 2	Negative
	Hepatitis A	Negative
	Hepatitis B	Negative
	Hepatitis C	Negative
	HIV	Negative
Flow cytometry lymphocyte subpopulations analysis	Regulatory *T* cells CD4 + CD25 ++ CD127−: 0.95% (>2%), without any other significant abnormalities.	
NGS*	NGS study of 192 genes related to immune dysregulation. No pathogenic or likely pathogenic variants were identified.	
Abdominal ultrasound	No significant findings	

* Genes studied: ACT1, ADA, AICDA, AIRE, AK2, AP3B1, AP3D1, ATM, BCL10, BLNK, BTK, CD3, CARD1, CARD9, CASP10, CASP8, CD127, CD19, CD20, CD21, CD27, CD3D, CD3E, CD3G, CD3Z, CD45, CD79A, CD79B, CD81, CD8A, CEBPE, CECR1, Cernunnos, CELC7A, COPA, CORO1A, CTLA4, CTPS1, CTSC, CXCR4, CYBA, CYBB, DCLRE1C, DKC1, DNMT3B, DOCK2, DOCK8, ELANE, EVER1, EVER2, FADD, FCGR3A, FOXN1, FOXP3, G6PC3, GATA2, GFI1, HAX1, HOIL1, ICOS, IFNGR1, IFNGR2, IGHM, IGLL1, IKZF1, IKBA, IKBKB, IKBKG, IL10, IL10RA, IL10RB, IL12B, IL12RB1, IL12RB2, IL17F, IL17RA, IL17RC, IL1RN, IL21, IL21R, IL2RA, IL2RG, IL7, IRAK4, IRF3, IRF7, IRF8, ISG15, ITGB2, ITK, JAGN1, JAK3, KIND3, KRAS, LAMTOR2, LCK, LIG4, LPIN2, LRBA, LYST, MAGT1, MALT1, MAP3K14, MCM4, MEFV, MHC2TA, MRE11, MST1MVK, MYD88, NCF1, NCF2, NFKB1, NFKBIA, NFKB2, NHP2, NLRC4, NLRP12, NLRP3, NOD2, NOP10, NRAS, ORAI1, p40phox, PGM3, PIKRCD, PIK3R1, PLCG2, PMS2, PNP, POLE1, PRF1, PRKCD, PSMB8, PSTPIP1, PTPN6, RAB27A, RAG1, RAG2, RFX5, RFXANK, RFXAP, RLTPR, RMRP, RNF168, RORC, RTEL1, SH2D1A, SMARCAL1, SP110, SPINK5, STAT1, STAT2, STAT3, STAT5B, STIM1, STX11, STXBP2, TAP1, TAP2, TAPBP, TBK1, TCF3, TCN2, TERC, TERT, TINF2, TIRAP, TLR3, TMEM173, TNFRSF13B, TNFRSF13C, TNFRSF1A, TNFRSF5, TNFRSF6, TNFSF5, TNFSF6, TRAF3, TRIF, TRNT1, TTC7A, TWEAK, UNC119, UNC13D, UNC93B1, UNG, VPS45, WAS, WIPF1, XIAP, XRCC4, ZAP70, ZBTB24.

A bone marrow study was performed to rule out other possible causes of anemia and thrombocytopenia, which revealed hypercellularity and megakaryocytic and erythroid hyperplasia, without any other abnormalities. A diagnosis of ITP and iron deficiency anemia was made. Treatment with intravenous immunoglobulins was started, without significant increases in platelet counts after 48 h. Corticosteroids were started due to platelet counts under 10 × 10^9 ^/L at a 2 mg per kg of body weight of prednisone dose for 5 days, with platelet counts rising to 70 × 10^9 ^/L on day 5. Iron supplements were started, and she was discharged from the hematology ward. However, at the 12-day follow-up, platelet counts dropped to 6 × 10^9^, leading to the reinstatement of corticosteroids at the same dose for another 5 days, which resulted in platelet counts reaching 100 × 10^9 ^/L by day 5. Similarly, hemoglobin levels returned to normal after 2 weeks of iron supplements treatment. This fast recovery could indicate an anemia of mixed origin, not only iron deficiency but also possibly related to a pro-inflammatory state in the context of new onset of ITP. The patient did not experience any significant bleeding episode during this period.

At discharge, she was referred to the Dermatology department, and she was diagnosed with alopecia areata, for which she received corticosteroids at a dose of 0.5 mg per kg of body weight of prednisone for a year with partial response. Platelet counts reached normal counts during this period. When corticosteroids were discontinued, alopecia worsened, platelet counts dropped below 100 × 10^9 ^/L, but no bleeding episodes occurred. At that time anti-microsomal autoantibodies, also known as anti-TPO antibodies, (8,547 UI/ml) were measured, without symptoms or any alteration on thyroid function studied by thyroid hormones. A screening for other autoimmune diseases was performed, with no significant findings (Table 1). Moreover, a next generation sequencing (NGS) panel of 192 genes related to immune dysregulation found no pathogenic or likely pathogenic variants. Baricitinib was then started off-label for the treatment of alopecia areata at a 4 mg/day dose, since its approval for that indication both by the Food and Drug Administration and the European Medicines Agency is limited to adult patients (3). Platelet counts when baricitinib was started were 70 × 10^9 ^/L, 2 years after the initial thrombocytopenia episode, leading to the diagnosis of chronic immune thrombocytopenia (cITP).

**Figure 1:**
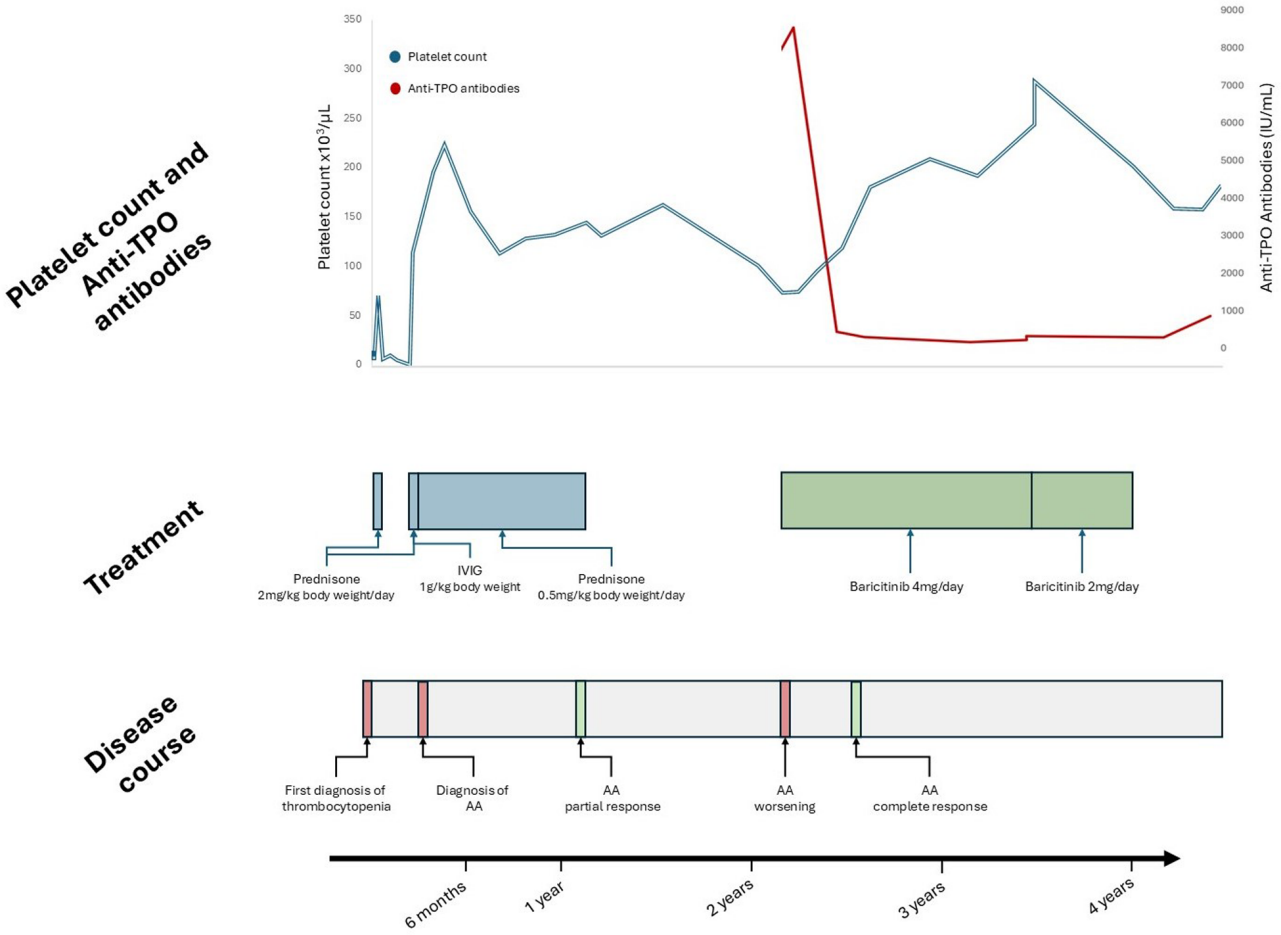
Patient follow-up. Anti-TPO antibodies, anti microsomal antibodies; IVIG, intravenous immunoglobulins; AA, alopecia Areata.

After 3 months of baricitinib treatment alopecia improved significantly. Treatment was well tolerated without any significant adverse effects. Platelet counts gradually increased, being higher than 150 × 10^9 ^/L five months later, and the anti-microsomal autoantibodies values significantly decreased from 8,547 IU/ml to 167.5 IU/ml one year later, without any other therapy. Lymphocyte phenotype was repeated under treatment, without any significant changes. After 18 months of treatment with baricitinib, the dose was reduced to 2 mg/day and discontinued after 6 months, with no significant decrease in platelet count and maintenance of hair regrowth achieved two years earlier. At 4 years follow-up, platelet counts remain within normal levels. Clinical course of the patient, platelet counts over time and treatments received are represented in Figure 1.

Written informed consent was obtained to publish this report.

## 3 Discussion and conclusions

As a JAK 1/2 inhibitor, baricitinib exerts a wide range of effects, modulating immune responses through the JAK/STAT pathway. It may increase platelet counts, although this is usually transient, with platelet counts returning to slightly above baseline levels. This effect may be related to an increase in thrombopoietin signaling due to selective JAK2 inhibition (4), to reduced destruction, reflected by a lower mean platelet volume (5), or both. Increasing evidence supports its use in adult patients in combination with high-dose dexamethasone as first-line therapy (6) and in corticosteroid-refractory cITP (7), with a median time to response of 12 days, and a median peak platelet count of 94 × 10^9 ^/L from a median baseline of 18 × 10^9 ^/L. Nevertheless, its efficacy in pediatric patients is unknown.

Baricitinib has been proven effective in other autoimmune conditions affecting pediatric patients (8). It is also effective in the treatment of alopecia areata in adults (2). Treatment of chronic autoimmune conditions represents a significant challenge. Furthermore, cITP, although less frequent in children than in adults (20%–30%) (9), may require several lines of treatment to achieve safe platelet levels.

Thyroid disease and ITP have long been known to be associated, and between 11.6% and 36% of pediatric ITP patients may develop antithyroid antibodies (10, 11). While previous studies suggested that pediatric ITP patients with positive antithyroid antibodies have lower platelet counts at diagnosis and a lesser response to intravenous immunoglobulins (10), recent reports did not find a correlation between the presence of these antibodies and a particular clinical phenotype or a higher risk of chronicity (11). Our patient had high titers of anti-TPO antibodies together with a slight decrease in platelet counts and a worsening of her alopecia areata, just before baricitinib treatment. These findings could be coincidental and the association of thyroid disease as a cause of a secondary ITP cannot be confirmed.

During baricitinib treatment our patient experienced an improvement in her other autoimmune condition, alopecia areata, and a significant reduction in anti-microsomal autoantibody levels. This suggests that the increase in platelet count could also be related to baricitinib treatment. As potential confounding factors, our patient may have experienced spontaneous ITP remission during baricitinib treatment, and platelet count fluctuations are also anticipated in ITP, even without any interventions, so the association between baricitinib treatment and platelet improvement remains speculative. The persistent remission after baricitinib was discontinued may indicate that it only had a suspensive effect and that our patient indeed underwent spontaneous remission. Our case may not be representative of the typical pediatric ITP, which usually presents itself at a younger age and without any other associated autoimmune conditions. Nevertheless, recent evidence from randomized clinical trials in adult patients suggest that baricitinib could potentially offer a novel and safe approach for treating chronic or refractory ITP. A subset of pediatric patients who could potentially benefit from baricitinib treatment includes those refractory to TPO-RA and those with other associated autoimmune diseases. A controlled clinical trial should be performed to assess the efficacy of baricitinib in this setting.

## Data Availability

The original contributions presented in the study are included in the article/Supplementary Material, further inquiries can be directed to the corresponding author.
